# Supplementary material for: Measuring the unknown: An estimator and simulation study for assessing case reporting during epidemics
Source: PLoS Comput Biol. 2022 May 23;18(5):e1008800. doi: 10.1371/journal.pcbi.1008800 (PMC9166360; doi:10.1371/journal.pcbi.1008800)

Measuring the unknown: an estimator and simulation study for assessing case reporting during epidemics

**Short title: Estimating reporting of cases in disease outbreaks**

Christopher I Jarvis^1,2^, Amy Gimma^1,2^, Flavio Finger^1,2,3^, Tim P Morris^4^, Jennifer A Thompson^1^, Olivier le Polain de Waroux^2,5^, W John Edmunds^1,2^, Sebastian Funk^1,2^,

Thibaut Jombart^1,2,6,7^

#

# Supplementary Tables

**Table A**. **Performance measures from 4000 simulation by the mean of the R distribution, reported outbreak size, and true reporting level.**


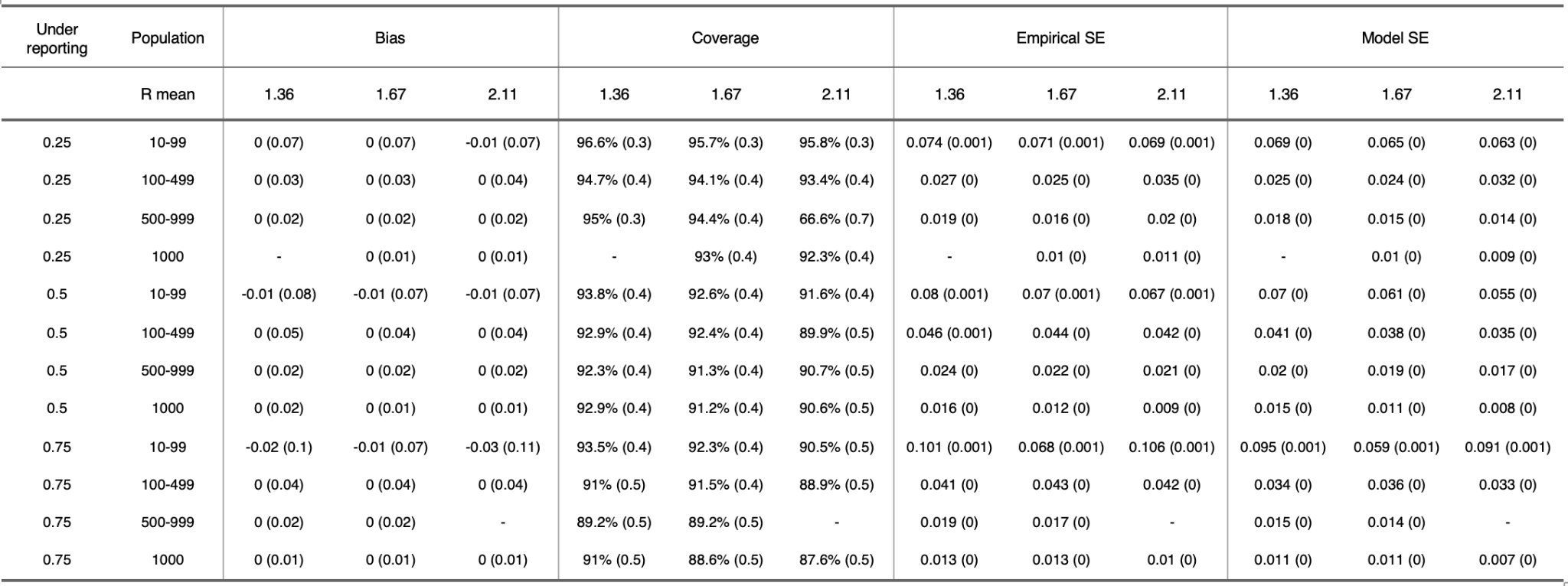


**Table B**. **Comparison of absolute error from 4000 simulations between true reporting levels and estimate of reporting by the mean of the R distribution, reported outbreak size, and true reporting level.**


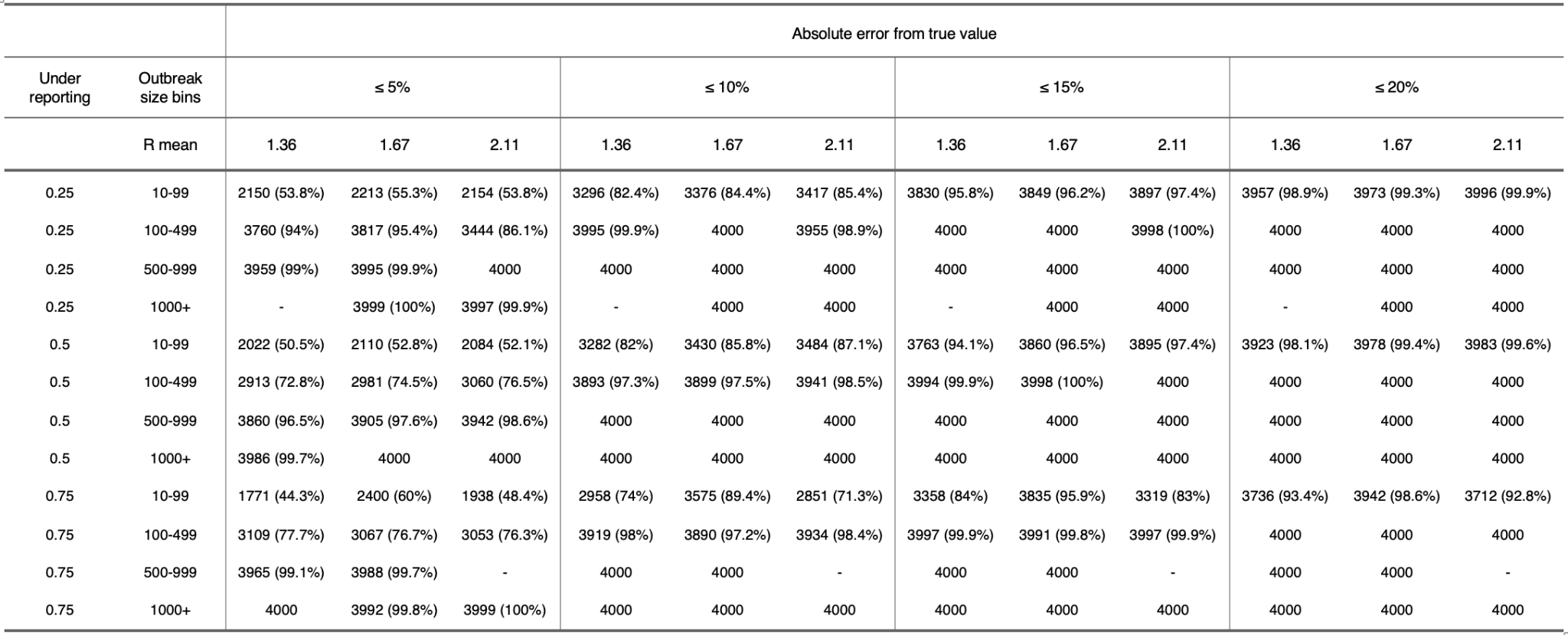

Supplement: S1 Text — Table A. Performance measures from 4000 simulation by the mean of the R distribution, reported outbreak size, and true reporting level. Table B. Comparison of absolute error from 4000 simulations between true reporting levels and estimate of reporting by the mean of the R distribution, reported outbreak size, and true reporting level. (DOCX) [file pcbi.1008800.s001.docx]
