# Supplementary material for: Measuring the unknown: An estimator and simulation study for assessing case reporting during epidemics
Source: PLoS Comput Biol. 2022 May 23;18(5):e1008800. doi: 10.1371/journal.pcbi.1008800 (PMC9166360; doi:10.1371/journal.pcbi.1008800)
Supplement: Attachment — Submitted filename: response letter.pdf [file pcbi.1008800.s002.pdf]

Dear Dr Jombart,

Thank you very much for submitting your manuscript "Measuring the unknown: an estimator and simulation study for assessing case reporting during epidemics" for consideration at PLOS Computational Biology.

As with all papers reviewed by the journal, your manuscript was reviewed by members of the editorial board and by several independent reviewers. In light of the reviews (below this email), we would like to invite the resubmission of a significantly-revised version that takes into account the reviewers' comments.

We cannot make any decision about publication until we have seen the revised manuscript and your response to the reviewers' comments. Your revised manuscript is also likely to be sent to reviewers for further evaluation.

When you are ready to resubmit, please upload the following:

[1] A letter containing a detailed list of your responses to the review comments and a description of the changes you have made in the manuscript. Please note while forming your response, if your article is accepted, you may have the opportunity to make the peer review history publicly available. The record will include editor decision letters (with reviews) and your responses to reviewer comments. If eligible, we will contact you to opt in or out.

[2] Two versions of the revised manuscript: one with either highlights or tracked changes denoting where the text has been changed; the other a clean version (uploaded as the manuscript file).

Important additional instructions are given below your reviewer comments.

Please prepare and submit your revised manuscript within 60 days. If you anticipate any delay, please let us know the expected resubmission date by replying to this email. Please note that revised manuscripts received after the 60-day due date may require evaluation and peer review similar to newly submitted manuscripts.

Thank you again for your submission. We hope that our editorial process has been constructive so far, and we welcome your feedback at any time. Please don't hesitate to contact us if you have any questions or comments.

Sincerely,

Benjamin Muir Althouse

Associate Editor

PLOS Computational Biology

Tom Britton

Deputy Editor

PLOS Computational Biology

Dear Editor,

Many thanks for your patience with this manuscript. The main authors and myself have been working full time on the COVID-19 response in the UK and internationally, and finding time for other work has been very challenging.

We thank both referees for their very thorough reviews of our work and insightful comments, which led, we believe, to an overall improvement of the manuscript. While we have not changed the method fundamentally, the reviews highlighted the need for clarification on several important points, particularly relating to the scope and applicability of the method. We have also added new simulations to provide a sensitivity study to assess the impact of different reproduction numbers on our estimates of reporting.

Below, you will find point-by-point responses (in black) to all comments (in grey); for convenience, we also added quotes of key changes in the manuscript (in italic; new content in blue).

Thank you for considering this revision.

Kind regards,

Thibaut Jombart

Reviewer's Responses to Questions

Comments to the Authors:

Please note here if the review is uploaded as an attachment.

Reviewer #1: The authors here seek to solve a critical problem in outbreak settings, the difficulty in understanding the reporting fraction in near-real time, without costly and time-consuming serological and genomic studies. The approach is simple (assuming extensive contact tracing and case investigation data exist) and intuitive, and the authors have structured the paper clearly.

My comments below seek to improve an approach for which I have high enthusiasm and believe could be very useful to the field. More clarity in the derivation/formalism and more extensive sensitivity analyses are need to show the validity of the approach, and several parts of the approach and simulation should be tailored to better address real-world outbreak scenarios (e.g., time-varying reporting, shorter time scale simulations).

1. I was confused whether this method accounts for undetected secondary cases. It seems the expression $E(n_u + n_k) = (m_r+m_K)*R$ would imply complete detection of the secondary cases. The expressions for $E(n_k)$ and $E(n_u)$, though, both have a pi term that I believe is account for under-reporting of secondary cases. Why is that not present in $E(n_u + n_k)$?

The referee is right, and the first expression is misleading - the correct definitions for $E(n_k)$ and $E(n_u)$ are the ones provided right after where ‘pi’ accounts for the reporting of the secondary cases. We have removed the first misleading expression, which was not useful in the proof.

Perhaps time indexing the estimator pi would help resolve some confusion. My understanding is that:

$pi_t = m_{r,t} / (m_{r,t} + m_{u,t})$

$n_{t+1} = (m_{r,t} + m_{u,t})*R$ (where n_{t+1} is the total number of secondary cases caused by index cases in generation t)

$n_{u, t+1} = m_{u,t}*R$

$n_{r, t+1} = m_{r,t}*R$

$n_{u, detected, t+1} = m_{u,t}*R*pi_{t+1}$

$n_{r, detected, t+1} = m_{r,t}*R*pi_{t+1}$,

where I've replaced n_u from the paper with $n_{u, detected, t+1}$, the number of detected secondary cases infected by an unknown infector of the previous generation, t, and n_k with $n_{r, detected, t+1}$ for the number of detected secondary cases infected by a known infector of generation t. The derivation then relies on the assumption that, at least over the interval of a generation time, $pi_t \sim pi_{t+1}$. Does this fit with the authors' logic? I think the assumption $pi_t \sim pi_{t+1}$ would be mostly valid in many scenarios, but should be explicitly stated.

We thank the referee for this suggestion. We had indeed considered time-indexing reporting in some earlier iterations of the method, but have decided against it for two main reasons: i) it made the formulation of the method substantially more complex, and ii) in practice, as the estimation of pi crucially depends on the number of cases successfully investigated over the time period considered, time-varying approaches (e.g. bi-weekly) would often suffer from low sample size and yield large uncertainty in the estimated pi and associated confidence intervals. Instead, we recommend using the method on fairly large time periods where reporting can be assumed to have been reasonably constant. We have now added this element to our discussion:

Simulation results indicate a first limitation of the method lies in the analysis of smaller outbreaks. Overall, the approach performed better in larger outbreaks, with all metrics pointing to improved results in outbreaks of more than 100 case investigations. This observation suggests that our method will often be unable to identify spatial variations in reporting at a fine spatial scale, where geographic units typically report few cases. Similarly, our approach is unlikely to be able to identify rapid changes in reporting over time, as short time spans (e.g. days or weeks) are unlikely to yield enough case investigations for an accurate estimation of reporting to be achievable.

See also the following response and quote, linked to this issue.

2. Time-indexing the reporting fraction would help indicate whether or how this method can be used to estimate a time-varying reporting fraction, as reporting fractions seem rarely to be constant (and cause the must frustration when they are not constant). This would be a critical expansion of the methods, and additional simulations with a non-constant reporting fraction would improve the paper.

This formalism could also help clarify to which time interval estimates apply. That is, should we consider pi_hat = n_k / (n_k + n_u), to use the original notation, as a lagged estimator by one generation time? How do reporting delays factor in? It's a fine point, but given recent discussion of cohort vs case reproductive numbers in this journal (https://doi.org/10.1371/journal.pcbi.1008409) and the relationship between time-varying reporting and R_t, perhaps of interest to the intended audience.

Thank you for this suggestion, which has helped us clarify our expectation of this approach. We appreciate the interest in estimating time-varying reporting, but as explained above and now in our discussion, we do not believe our methodology is best-suited for that purpose. Indeed, the need for a substantial number of case investigations (~100) means the time period considered needs to be sufficiently large to reach these numbers, and it is highly unlikely we could estimate daily or even weekly variations in reporting as is done e.g. with the instantaneous reproduction number. Note that in terms of outbreak response, decisions for altering surveillance strategies would generally be made at coarse geographic scales and considering months of data, so that tracking the reporting day by day or week by week would be of less issue.

We have added the following to the discussion:

As a consequence, our method is best applied for estimating the overall reporting over geographic areas and time periods where surveillance has not varied drastically, and which are large enough to yield sufficient case investigations (typically at least 100) for reporting to be accurately estimated. Note that in terms of outbreak response, decisions for altering surveillance strategies would generally be made at coarse geographic scales and considering months of data, so that our method should retain operational value despite its inability to detect changes in reporting at small temporal or spatial scales.

3. I am concerned about the situation where an infector is reported, but not known to be the infector of a given case. That is, what happens when the assumption that "all cases reported were investigated, so that it is known if they had a documented epidemiological link, or not, amongst reported cases" breaks down? I understand this may be less common in the context of EVD, but seems more likely in the case of COVID, for example, for which this method would be very useful. In this situation, the unknown but detected infector would lead to inflation of the denominator (i.e., over-counting of n_u) and underestimation of pi. Some discussion or simulation (e.g. by pruning known transmission links, not solely index cases) would be useful.

We agree with the referee, and have incorporated this point to our discussion:

As our method performs better in larger datasets, the data requirement for estimating reporting from transmission chains will involve substantial field work. This also implies that case investigations need to be thorough. Indeed, in situations where the infector has actually been reported, but investigations failed to identify the epidemiological links with their secondary cases, our approach will tend to under-estimate reporting by a factor directly proportional to the frequency of mis-identified links. As a consequence, the proposed methodology is mostly applicable to diseases for which person-to-person transmission can be achieved through epidemiological investigation such as EVD, and may be less useful for diseases such as COVID-19, where transmission chains are harder to establish.

This would be a larger expansion, but it does seem like it would be possible to incorporate some probabilistic reconstruction (rather than just known/unknown links) to account for scenarios of several likely infectors, or an unknown infector, as described in the discussion.

We agree a probabilistic approach to reconstructing transmission trees is also an interesting take on the problem, but it also strays slightly from the approach we propose. It would be more in line with methods such as the models implemented in the R package *outbreaker* 2, developed by the last author of the present work, which provides a Bayesian framework for inferring who infected whom, and can also estimate reporting [1–3] . This type of approach is discussed in our discussion, but is vastly more complex than the proposed method, and has even stronger data requirements. In practice, our experience suggests such methods are still rarely used to inform decision-making in real time, and we believe the simplicity of the proposed approach and its use of already routinely collected data make it more applicable in the field.

4. I can't speak as much to the theoretical validity of using standard errors for a proportion and exact binomial confidence intervals, though I am concerned by the low coverage. My two primary concerns, both partially addressed in the limitations, are (1) possible links/correlation of detection in a transmission chain (i.e., a secondary case of an undetected index is less likely to be detected itself; it would seem this might have less influence on uncertainty and may be more of a structural issue, that pi_{t+1} depends on m_{u,t} vs m_{r,t}) and (2) when there is relationship between R and pi (e.g., super-spreading events more likely to be detected), or even just overdispersion in R leading to larger variance in n and hence pi.The discussion does partially address #1, saying the approach would "be prone to under-estimating reporting when entire branches of the transmission tree remain unobserved"; but what if there is not complete non-report of certain chains, and just systematic under-reporting?

Our approach does not distinguish between different transmission chains. In the situation described by the referee , the outcome would be an average of the two branches mentioned, weighted by the number of successfully investigated cases with a known infector in each branch. We have added the following to the discussion:

Similarly, the fact that a single overall value of reporting is estimated for the data considered also implies that changes in reporting across different transmission chains will be overlooked. In situations where reporting varies across chains, for instance if super-spreading events are systematically 'better' investigated, the estimated reporting would effectively be an average of the reporting levels of the different chains weighted by their respective numbers of successful case investigations.

Similarly, the authors note that if super-spreaders were more likely to be undetected, "we would expect our method to under-estimate reporting, although this should be further quantified by dedicated simulation studies". Are the authors not able to perform these simulations? Does simulacr allow for negative binomial branching?

The presentation of our simulations lacked clarity, as we are indeed implicitly using a NegBin branching process. Indeed, the reproduction numbers are Poisson-distributed, but the rates of the Poisson themselves are Gamma-distributed with a rate of 1.2 and shape of 2, which is equivalent to a negative binomial process. So we do allow for superspreading to take place in our simulations, but we did not estimate a specific impact of super-spreading on our approach. For such an assessment to be made, we would have needed to control the level of super-spreading and treated it as an additional simulation parameter. We aimed to keep the number of varying aspects of the simulations to a few to explore the statistical properties of the estimator before exploring further real world considerations in future work.

We have clarified the method section:
In its basic form, simulacr implements a Poisson branching process in which the reproduction numbers (R) is combined with the infectious period to determine individual rates of infection. Here, to account for potential heterogeneity in transmission, we have drawn individual values of R from a Gamma distribution fitted to empirical data from the North Kivu EVD epidemic (rate: 1.2; shape: 2; corresponding mean: 1.7). The resulting branching process being a combination of Poisson processes with Gamma-distributed rates is therefore a Negative Binomial branching process.

5. The definitions of bias and coverage are very clear. I was confused, though, by the statement that "the model based standard error is mean of the square of the bias", when Table 2 lists the model based standard error as the square root of Var(pi). How are the authors defining Var(pi) (presumably distinct from the standard error of pi_hat on pg 6?)

We thank the referee for pointing out this issue. Table 2 had the definition of the model based and the empirical standard error swapped over. We have now updated the model based standard error to be $E[(\widehat{\theta} - \theta)^2]$. Var(pi) was also supposed to be $\sqrt{Var(\widehat{\theta})}$ and has now been updated for the empirical standard error. We have taken these definitions from Table 6 in Morris et al 2019 https://onlinelibrary.wiley.com/doi/10.1002/sim.8086

6. It seems all models were run for 365 days with a fixed distribution of R, and thus it was differences in population size and reporting that dictated reported outbreak size. It would greatly improve the paper to see the patterns in bias/precision broken down by R and time scale, not just a proxy of population size. That is, does a reported outbreak of n=100 across one year due to low R and small population behave the same as a reported outbreak of n=100 across one month with higher R? Exploring these shorter time frames would seem to be highly relevant for the outbreak scenarios in which this method would be applied. I also suspect in short time scales and smaller populations are where differences in a Poisson vs negative binomial branching process (or even just differences in the distribution of R) could become most important in appropriately considering uncertainty.

We agree, and have generated new results to address this point, varying the rates of the Gamma distribution to increase the range of *R* values across all simulations. We evaluated the impact of different average values of *R* thus obtained (1.4, 1.7, 2.1) on the estimation of reporting. Results on the different performance metrics are presented in the new Supplementary Tables 1 and 2. In short, it looks like the metrics are overall unaffected by the value of *R*, and we see similar issues with coverage for different values of *R*.

We have add new sections in the Methods and Results, accordingly, as well as 2 supplementary tables:

Sensitivity to R values

In order to explore the sensitivity of the method to the distribution of R, we repeated the simulations and analyses using different distributions of the reproduction number, using Gamma(rate = 0.95, shape = 2) and Gamma(rate = 1.475, shape = 2), resulting in average R values of 2.1 and 1.3, respectively, broadly in line with values reported in the literature for other EVD outbreaks [4].

Sensitivity to *R* values

Repeating the analyses with different *R* distributions in line with reproduction numbers reported in other outbreaks [4] showed negligible impact of *R* on bias, coverage, precision,

and absolute error (Supplementary Tables, S1 and S2). Coverage was the most sensitive to the change in *R*, which slightly decreased with higher mean *R* values. Overall though, variations were negligible compared to variations of coverage with epidemic size.

Reviewer #2: The authors describe a novel method for estimating the proportion of cases that are observed during an epidemic. Overall the manuscript is well presented and the methods and results described clearly.

I raise (major) points below about the appropriateness of the measure used to estimate reporting (cases with "known" infectors) and the generalisability of their findings across outbreaks of different pathogens.

1. The key measure from the epidemiological data used is "cases with a known infector". I initially looked for information on how a "known infector" is classified in the Methods, but found it later in the Discussion. "Our approach relies on case investigation data, a time-consuming process usually requiring interviews of patients and/or their close relatives. There are several possible outcomes from such investigation: i) a single infector can be identified..." etc

As this definition and the description of the underlying data is important it should be visible much earlier in the paper.

We agree and have added this definition at the beginning of the methods section:
Estimating reporting from epidemiological links

Our method exploits transmission chains derived from case investigation and contact tracing data. The data considered are secondary cases for which epidemiological investigation was successfully carried out and either i) identified an infector amongst reported cases (cases with a known infector) or ii) established that the infector was amongst the reported cases (cases without a known infector).

My impression is that determining "who infected whom" is difficult in the absence of genomic data. Contact tracing methods may be effective when a pathogen is "rare", i.e. a primary infection occurs in a village in a person who has travelled from outside, and then a second case is reported in the same household within the serial interval. But such examples are likely rare? When transmission is widespread and there are multiple transmission chains circulating simultaneously, inferring who infects whom is very difficult. Therefore the accuracy of interviews to determine infector/ infectee pairs is likely to decrease as the outbreak size increases. Do the authors agree that this is a potential source of bias that should be accounted for in their simulations?

We agree that reconstructing transmission chains in the general case is a difficult problem, which we have worked on separately [1–3,5]. While it seems like identifying transmission chains should become increasingly difficult with the outbreak size, our experience in the EVD North Kivu outbreak was different. Indeed, while cases go up, the exposure of patients

to potential infectors usually remains constant: the outbreak spreads by contaminating new households, districts and villages, but cases of multiple exposures remain infrequent, and failures to identify the infector usually stems from other factors (patients lying about exposure, or inability to communicate with the patients or close contacts).

We do acknowledge though that it is important to define when the method is applicable. We added to the discussion (also addressing comments of referee 1):

As our method performs better in larger datasets, the data requirement for estimating reporting from transmission chains will involve substantial field work. This also implies that case investigations need to be thorough. Indeed, in situations where the infector has actually been reported, but investigations failed to identify the epidemiological links with their secondary cases, our approach will tend to under-estimate reporting by a factor directly proportional to the frequency of mis-identified links. As a consequence, the proposed methodology is mostly applicable to diseases for which person-to-person transmission can be reconstructed through epidemiological investigation such as EVD. In disease settings where transmission chains are harder to establish, such as COVID-19 where pre-symptomatic and asymptomatic transmission plays an important role, we recommend resorting to other surveillance approaches such as serological surveys to estimate reporting.

2. It is implied that the method is generalisable across outbreaks with different pathogens. In using data only from a single pathogen in a single location, however, this claim is suspect and not proven. Could simulations be performed which are calibrated to the natural history of other pathogens? In particular, I wonder if the method would be successful at inferring the level of reporting in infections with a high proportion of asymptomatic cases or with a variable serial interval. In both of these cases, I suspect it would be more difficult to correctly assign infector/ infectee pairs.

We agree, and tried to clarify the scope of applicability of the method (see response to previous point). Indeed, more simulations for different pathogens would be useful to focus on aspects that impact the approach. We have emphasized that the approach is at present more Ebola-focused and that alternatives such as sero surveys should be considered when transmission chains are less clear-cut.

3. The underlying model does not account for an over-dispersed distribution of secondary cases (negative binomial distribution, or "super-spreading"). Although this is mentioned as a limitation in the Discussion, it does raise questions about the appropriateness of the epidemiological model, given that overdispersion in the secondary case distribution is a key driver of epidemics. There are estimates of the dispersion parameter k for Ebola virus (e.g. 0.37 in Lau et al. PNAS 2017), therefore the authors could use this information in their model. For instance, if one person is identified as an infector, what is the probability that they did indeed only infect one other person? When k is small, this probability is low and there are likely several other cases non-reported.

This echoes previous points by referee 1. The description of the model was unclear: we do, in fact, use a Negative Binomial branching process, as we use a mixture of Poisson with Gamma-distributed reproduction numbers. This has been clarified in the method section (see response to referee 1). We have also added new simulations to evaluate the impact of different distributions of *R*, and found that overall, this had a minimal impact on the results.

4. In the introduction the authors characterise alternative measures to estimate the reporting rate as difficult or impractical, although the data collecting method for their own analysis is later described as "a time-consuming process". There is no mention of prevalence surveys to estimate the true number of cases - e.g. the REACT study which is ongoing in the UK for COVID-19. How would (a small number of) cluster-randomised prevalence surveys perform as a method to estimate reporting, given that interviewing cases is "time consuming", and likely subject to bias (see point 1)?

We agree, but would argue that differences in epidemic settings will in practice have a very strong impact on the optimal methodology. Sero-surveys such as REACT are indeed extremely valuable, but also near-impossible to put in place in resource-limited contexts and fragile environments, where the impact of the response on local populations needs to be minimized. In the case of EVD, case investigation and contact tracing, while indeed labour intensive, are standard approaches of the response as they are used for improving case detection and for ring vaccination.

This is now mentioned in the discussion:

As a consequence, the proposed methodology is mostly applicable to diseases for which person-to-person transmission can be reconstructed through epidemiological investigation such as EVD. In disease settings where transmission chains are harder to establish, such as COVID-19 where pre-symptomatic and asymptomatic transmission plays an important role, we recommend resorting to other surveillance approaches such as serological surveys to estimate reporting.

1. Jombart T, Cori A, Didelot X, Cauchemez S, Fraser C, Ferguson N. Bayesian reconstruction of disease outbreaks by combining epidemiologic and genomic data. PLoS Comput Biol. 2014;10: e1003457.
2. Campbell F, Cori A, Ferguson N, Jombart T. Bayesian inference of transmission chains using timing of symptoms, pathogen genomes and contact data. PLoS Comput Biol. 2019;15: e1006930.
3. Campbell F, Didelot X, Fitzjohn R, Ferguson N, Cori A, Jombart T. outbreaker2: a modular platform for outbreak reconstruction. BMC Bioinformatics. 2018;19: 363.
4. Van Kerkhove MD, Bento AI, Mills HL, Ferguson NM, Donnelly CA. A review of epidemiological parameters from Ebola outbreaks to inform early public health decision-making. Sci Data. 2015;2: 150019.
5. Campbell F, Strang C, Ferguson N, Cori A, Jombart T. When are pathogen genome sequences informative of transmission events? PLoS Pathog. 2018;14: e1006885.
