# Supplementary material for: Measuring the unknown: An estimator and simulation study for assessing case reporting during epidemics
Source: PLoS Comput Biol. 2022 May 23;18(5):e1008800. doi: 10.1371/journal.pcbi.1008800 (PMC9166360; doi:10.1371/journal.pcbi.1008800)
Supplement: Attachment — Submitted filename: reporting_response letter_v2.docx [file pcbi.1008800.s003.docx]

Dear Editor,

Many thanks for allowing us to revise our manuscript. Apologies again for the delays in finalising this work, due to our involvement in the COVID-19 response in the UK and internationally.

We thank both referees for their useful comments. In this revision, we have clarified the points raised in the reviews and fixed issues raised by the referees. We were unable to add analyses of the North Kivu Ebola outbreak recommended by referee 2: while we agree this would be a nice illustration for the method, we have no data sharing agreement for publishing results using these data, and putting one in place amidst the COVID-19 crisis would take months.

Below, you will find point-by-point responses (in black) to all comments (in grey); for convenience, we also added quotes of key changes in the manuscript (in italic; new content in blue).

Thank you for considering this revision.

Kind regards,

Christopher Jarvis

Reviewer's Responses to Questions

**Comments to the Authors:**

**Please note here if the review is uploaded as an attachment.**

Reviewer #1: I thank the reviewers for their thorough responses to my comments. This paper would continue to benefit from refinement of how the methods and its valid applications are presented, as discussed below.

1. I still find there to be a bit of an internal conflict in the described applications of this method. The method is described in the abstract and introduction as "useful for estimating reporting in real-time" and as a tool that can be used "to inform strategic decision making during an outbreak response". The proposed use case in the author's response, however, is to guide decisions for altering surveillance strategies using "months of data", rendering concerns about temporal and spatial variations of lesser importance. To me, that's not exactly a 'real-time' application and doesn't allow for operational results in the critical early weeks or months (depending on case rate) of an outbreak. Relatedly, while I think a popular use of reporting fraction estimates is to understand current burden (think of the extensive "nowcasting" COVID literature), this method would appear to be ill-suited except in the case that extensive investigation of multiple parallel transmission chains can be completed quickly.

Clarifying the valid uses of this method earlier in the paper, specifically the sample size limitations and associated limitations in temporal and spatial resolution and the bias from incomplete tracing, would improve the framing of this paper. I would soften language in the introduction, particularly in paragraph 2 when describing "timely estimation" amidst changing outbreak situations.

Response: We agree with the referee on the need for clarifying use-cases of the method.

The introduction now puts less emphasis on rapid estimation:

*Therefore, simpler approaches are needed for estimating reporting and help inform outbreak response operations.*

*Methods for estimating reporting during an outbreak should ideally exploit data which is routinely collected as part of the outbreak response.*

*The second paragraph of the discussion now reads:*

*Overall, the approach performed better in larger outbreaks, with all metrics pointing to improved results in outbreaks of more than 100 case investigations. This observation suggests that our method may struggle to identify heterogeneities in reporting across time, space, or sections of the population (e.g. age groups) if the corresponding strata have small numbers of reported cases. It also means that estimates of reporting made in the early stages of an outbreak, when few cases have been reported and investigated, will be prone to larger statistical uncertainty.*

2. Thank you to the authors for providing the reference for their definitions of model based standard error and empirical standard error. The definition of the empirical SE is now more clear. I am still confused, though, by the definition of the model-based standard error. The definition E[(theta_hat - theta)^2] given in Table 3 is the classical definition of the mean squared error; this is also the name used in Table 6 of Morris et al. The written definition that the model-based square error (the MSE) is "the mean of the square of the bias" is confusing, in that in that MSE = E[Bias_i^2], if Bias_i = theta_{hat,i} - theta, but MSE!=Bias^2, when Bias = E(theta_hat)-theta, as defined earlier in Table 3. Did the authors intend to use a different quantity than the MSE? If not, it would be more clear and better match common terminology to refer to the model-based square error as the MSE.

If the model standard error is in fact the MSE, I would expect MSE = Var(theta_hat) + Bias^2, where Bias = E(theta_hat)-theta. This relationship does not appear to hold in Table 3 (e.g., for pi=0.25 and outbreak size 10-99, I would expect MSE ~ 0.071^2 = 0.005). The derived quantity in the authors' code may be incorrect. The model based standard error (`mod_se_bias`, or `model_se` as used in Table 3) appears to be the square root of the mean across all simulations of the squared reporting probability standard errors [that is, sqrt( mean( reporting_probability * (1 - reporting_probability) / n_reported ) )]. What is defined as `rmse` or `root_mean_squared_error` appears to be correct [that is, sqrt( mean( (reporting_fraction - true_value)^2 ) )], but does not appear to be used in Table 3.

Response: Thank you for spotting this and we apologise for the confusion, you are correct. We intended to use the model-based standard error (and this is what was calculated in the code) but we described and defined the mean squared error in the text. It should have been the model-based standard error.

We have changed the formula to be $\sqrt{E[\hat{Var}(\hat{\theta})}]$ and have changed the word description to the following:

*The model based standard error is the root of the mean estimated variance*

3. Do the authors mean "ii) established that the infector was NOT amongst the reported cases (cases without a known infector)."? I think it is worth addressing the difference between establishing that the infector was not amongst reported cases and being unable to establish whether the infector is among reported cases; the former implies more certainty than I think there often is in the case of unidentified infectors.

Response: We agree this is an important clarification, and have incorporated the suggestion in our discussion:

*There are several possible outcomes from such investigation: i) identifying a single likely infector amongst reported cases (cases with a known infector) ii) establishing that the infector was not amongst the reported cases (cases without a known infector) iii) failing to identify a single likely infector. Our approach requires case investigations to fall within the first two categories. In practice, the second and third situations maye be easily confused - the third likely being the most frequent case. To avoid such confusion, we would recommend recording outcomes as two separate questions: can a single likely infector be identified? And if yes, is this individual listed amongst reported cases?*

4. The sentence "As a consequence, the proposed methodology is mostly applicable to diseases for which person-to-person transmission can be achieved through epidemiological investigation such as EVD" is unclear; do the authors mean that "person-to-person transmission can be reliably traced" or similar? This is a critical assumption that merits more treatment in the methods (e.g., following the definitions of cases with/without known infectors in "Estimating reporting from epidemiological links").

Response: We rephrased as suggested:

As a consequence, the proposed methodology is mostly applicable to diseases for which person-to-person transmission can be reliably traced through epidemiological investigation such as EVD.

We have also added some clarification to the method section, at the recommended place:

*The data considered are secondary cases for which epidemiological investigation was successfully carried out, and for which a single likely infector could be clearly identified. We thus distinguish i) cases for which the identified infector is listed amongst reported cases (cases with a known infector) and ii) cases for which the identified infector is not listed amongst the reported cases (cases without a known infector). Importantly, cases without any known exposure, or cases for which multiple epidemiological links make it hard to disentangle a single likely infector, are excluded from the analysis.*

5. I would explicitly state in the derivation that reported and unreported infectors are assumed to have same distribution of R (or do the authors believe that only the average R must be constant, in which case this should be stated).

Response: Thank you for this suggestion. This has now been added to the text:

$R$ - reproduction number, i.e. average number of secondary cases by case; we assume reported and unreported infectors have the same distribution of *R*

6. I believe the definition of the exact (Clopper Pearson) interval includes the multiplier (n_k + 1) in the denominator of the second term, rather than n_k alone, for the upper bound. The correct definition was implemented in the code.

Response: Thank you for spotting this, you are correct, and we have updated the definition in the manuscript accordingly. Thank you for also checking the implementation in the code.

7. Code for reproducing simulations and manuscript figures is generally well commented. This could be user error, but I was unable to run the parallelized outbreak simulations without adding the pkg:: operator whenever `simulacr` functions were required (simulacr::make_disc_gamma in create_raw_simulation_list and simulacr::simulate_outbreak in recursive_minimum_outbreak, specifically). Better documentation for which version/branch to use and/or a code release would be helpful.

Response: Thank you for pointing this out. We are not certain why this is as the scripts 01_simulate_outbreaks, 02_remove_cases, load the simulacr package at the top so it shouldn’t require the additional code inside the functions. But just to make sure, we have added in explicit reference to the simulacr functions.

We have also added a script 00_packages which will install the needed packages, and updated the documentation. Thank you for checking the code, and taking the time to troubleshoot the solution, this is really appreciated.

Reviewer #2: The authors have provided robust responses to the reviewer comments and made modest alterations to their manuscript.

My remaining concern is that the method has not been applied to estimate reporting in the original EVD dataset. To pose as a question - what was the fraction of cases reported during the EVD epidemic in DRC? Without this application the manuscript feels incomplete, particularly as there are references in both the paper and the response to reviewers that this study is EVD focussed rather than a generalisable tool. It also undermines the point made in the Introduction that the fraction of cases reported is a "key epidemiological indicator" and an "essential factor to consider". If reporting the level of reporting is so important, then why not report it? As the method is "fast and simple" this should presumably not take long.

In addition, some context on the original dataset would be beneficial. There are pointers to other studies, but the Methods should include, as a minimum, the locations where contact tracing data were collected, the dates of collection, and by whom they were collected (Public health ministry/ NGO). Ideally a template of the interview form used for contact tracing should be included as supplementary information.

Response: We agree with the referee that estimates of reporting using the North Kivu outbreak data would be a good illustration for the method, in addition to the analyses of simulated outbreaks. Unfortunately, we will be unable to provide this illustration. Indeed, we are lacking a data sharing agreement for publishing these results, and obtaining one amidst the COVID-19 crisis would take months (if at all possible), as all our former colleagues from the Ministry of Health in DRC, and at the WHO, are swamped with COVID-19 work.

For the same reason, we will not be able to share case investigation forms. We agree that recording the information needed by the method as accurately as possible is an important issue, and indeed, the confusion between ‘no information on a single infector’ and ‘the infector is identified, but not listed in the case line lists’ is one we have experienced, and which needs clarifying. In line with this comment, and another comment from referee 1, we have now clarified this point in our discussion:

*Our approach relies on case investigation data, a time-consuming but often standard process of contact tracing usually requiring interviews of patients and/or their close relatives. There are several possible outcomes from such investigation: i) identifying a single likely infector amongst reported cases (cases with a known infector) ii) establishing that the infector was not amongst the reported cases (cases without a known infector) iii) failing to identify a single likely infector. Our approach requires case investigations to fall within the first two categories. In practice, the second and third situations may be easily confused - the third likely being the most frequent. To avoid such confusion, we would recommend recording investigation outcomes as two separate questions: Has a single likely infector been identified? And if yes, is this individual listed amongst reported cases?*

**Have the authors made all data and (if applicable) computational code underlying the findings in their manuscript fully available?**

The [PLOS Data policy](https://protect-eu.mimecast.com/s/fMBzCWqVNfyDkm1S6UZ8X?domain=journals.plos.org) requires authors to make all data and code underlying the findings described in their manuscript fully available without restriction, with rare exception (please refer to the Data Availability Statement in the manuscript PDF file). The data and code should be provided as part of the manuscript or its supporting information, or deposited to a public repository. For example, in addition to summary statistics, the data points behind means, medians and variance measures should be available. If there are restrictions on publicly sharing data or code —e.g. participant privacy or use of data from a third party—those must be specified.

Reviewer #1: Yes

Reviewer #2: None

PLOS authors have the option to publish the peer review history of their article ([what does this mean?](https://protect-eu.mimecast.com/s/ZzqbCX5WOIMOJoltVzXxH?domain=journals.plos.org)). If published, this will include your full peer review and any attached files.

If you choose “no”, your identity will remain anonymous but your review may still be made public.

**Do you want your identity to be public for this peer review?** For information about this choice, including consent withdrawal, please see our [Privacy Policy](https://protect-eu.mimecast.com/s/XrdOCY58PI6A82KC9NvQY?domain=plos.org).

Reviewer #1: No

Reviewer #2: No

Figure Files:

While revising your submission, please upload your figure files to the Preflight Analysis and Conversion Engine (PACE) digital diagnostic tool, [https://pacev2.apexcovantage.com](https://protect-eu.mimecast.com/s/5Vy9CZ01QioQG91Uy6KBA?domain=pacev2.apexcovantage.com). PACE helps ensure that figures meet PLOS requirements. To use PACE, you must first register as a user. Then, login and navigate to the UPLOAD tab, where you will find detailed instructions on how to use the tool. If you encounter any issues or have any questions when using PACE, please email us at figures@plos.org.

Data Requirements:

Please note that, as a condition of publication, PLOS' data policy requires that you make available all data used to draw the conclusions outlined in your manuscript. Data must be deposited in an appropriate repository, included within the body of the manuscript, or uploaded as supporting information. This includes all numerical values that were used to generate graphs, histograms etc.. For an example in PLOS Biology see here: [http://www.plosbiology.org/article/info:doi/10.1371/journal.pbio.1001908#s5](http://protect-eu.mimecast.com/s/2J6xC14LACEqRNrUYvrN-?domain=plosbiology.org).

Reproducibility:

To enhance the reproducibility of your results, we recommend that you deposit your laboratory protocols in protocols.io, where a protocol can be assigned its own identifier (DOI) such that it can be cited independently in the future. Additionally, PLOS ONE offers an option to publish peer-reviewed clinical study protocols. Read more information on sharing protocols at [https://plos.org/protocols?utm_medium=editorial-email&utm_source=authorletters&utm_campaign=protocols](https://protect-eu.mimecast.com/s/kVkNC2WMBTE8jzLUMd1uo?domain=plos.org)
